# Improved quality of physiotherapy care in patients with Whiplash-Associated Disorders: Results based on 16 years of routinely collected data

**DOI:** 10.3389/fpain.2022.929385

**Published:** 2022-08-30

**Authors:** Rob A. B. Oostendorp, Hans Elvers, Emiel van Trijffel, Geert M. Rutten, Gwendolyne G. M. Scholten-Peeters, Margot De Kooning, Marjan Laekeman, Jo Nijs, Nathalie Roussel, Han Samwel

**Affiliations:** ^1^Scientific Institute for Quality of Healthcare, Radboud University Nijmegen Medical Centre, Nijmegen, Netherlands; ^2^Department of Manual Therapy, Faculty of Medicine and Pharmacy, Vrije Universiteit Brussel, Brussels, Belgium; ^3^Pain in Motion International Research Group, Vrije Universiteit Brussel, Brussels, Belgium; ^4^Practice Physiotherapy and Manual Therapy, Heeswijk-Dinther, Netherlands; ^5^Department of Public Health and Research, Radboud University Nijmegen Medical Centre, Nijmegen, Netherlands; ^6^Methodological Health-Skilled Institute, Beuningen, Netherlands; ^7^SOMT University of Physiotherapy, Amersfoort, Netherlands; ^8^Department of Physiotherapy, Human Physiology and Anatomy, Faculty of Physical Education and Physiotherapy, Vrije Universiteit Brussel, Brussels, Belgium; ^9^Ziekenhuisgroep Twente, ZGT Academy, Almelo, Netherlands; ^10^Research Program of Organization of Healthcare and Social Services, School of Health Studies, HAN University of Applied Science, Nijmegen, Netherlands; ^11^Department of Human Movement Sciences, Faculty of Behavioral and Movement Sciences, Vrije Universiteit Amsterdam, Amsterdam Movement Sciences, Amsterdam, Netherlands; ^12^Department of Physical Medicine and Physiotherapy, University Hospital Brussels, Brussels, Belgium; ^13^Department of Physiological Psychology, Otto-Friedrich University of Bamberg, Bamberg, Germany; ^14^Department of Physiotherapy and Rehabilitation Sciences (MOVANT), Faculty of Medicine and Health Sciences, University of Antwerp, Antwerp, Belgium; ^15^Integrated Health Care Clinics (IHC), 's-Hertogenbosch, Netherlands

**Keywords:** quality indicators, clinical reasoning, routinely collected data, clinical registry data, physiotherapy, quality improvement, whiplash injury, implementation science

## Abstract

Quality improvement is now a central tenet in physiotherapy care, and quality indicators (QIs), as measurable elements of care, have been applied to analyze and evaluate the quality of physiotherapy care over the past two decades. QIs, based on Donabedian's model of quality of care, provide a foundation for measuring (improvements in) quality of physiotherapy care, providing insight into the many remaining evidentiary gaps concerning diagnostics, prognostics and treatment, as well as patient-related outcome measures. In this overview we provide a synthesis of four recently published articles from our project group on the topic of quantitative measures of quality improvement in physiotherapy care, in this context specifically focused on patients with WAD in primary care physiotherapy. A set of process and outcome QIs (*n* = 28) was developed for patients with WAD and linked to a database consisting of routinely collected data (RCD) on patients with WAD collected over a 16-year period. The QIs were then embedded per step of the clinical reasoning process: (a) administration (*n* = 2); (b) history taking (*n* = 7); (c) objectives of examination (*n* = 1); (d) clinical examination (*n* = 5); (e) analysis and conclusion (*n* = 1); (f) treatment plan (*n* = 3); (g) treatment (*n* = 2); (h) evaluation (*n* = 5); and (i) discharge (*n* = 2). QIs were expressed as percentages, allowing target performance levels to be defined ≥70% or ≤30%, depending on whether the desired performance required an initially high or low QI score. Using RCD data on primary care patients with WAD (*N* = 810) and a set of QIs, we found that the quality of physiotherapy care has improved substantially over a 16-year period. This conclusion was based on QIs meeting predetermined performance targets of ≥70% or ≤30%. Twenty-three indicators met the target criterium of ≥70% and three indicators ≤30%. Our recommended set of QIs, embedded in a clinical reasoning process for patients with WAD, can now be used as a basis for the development of a validated QI set that effectively measures quality (improvement) of primary care physiotherapy in patients with WAD.

## Introduction

Quality improvement is no longer the preserve of a few enthusiastic professionals but has become a central tenet in healthcare, including physiotherapy. Quality improvement is now part of the daily routine of all those involved in delivering healthcare and is even a statutory obligation in many countries (1), including the Netherlands (2). Physiotherapists are directly or indirectly involved in optimal physiotherapy care not only in hospitals but also increasingly in primary care. Measurement of quality using quality indicators (QIs) plays an important role in improvement of healthcare (3–6).

Clinical registries, including routinely collected data (RCD), are recognized as an important source of data and harbor the potential to improve quality of care (7). They provide data about variation in quality of care, whether benchmarks are being met and facilitate feedback to clinicians, managers, funders, policymakers and researchers. Using clinical registries to inform data-driven quality improvement projects has resulted in the promotion of best practice and further stimulated use of registry data for quality improvement (7). Physiotherapists have monitored quality of care since the 1990's. During workshops in 1992, in which the methodology of indicator development for physiotherapy was explored, the Australian Physiotherapy Association adopted the concept of QIs to measure the quality of physiotherapy care (8). Around the same period (1990), the project “Quality in Physiotherapy” was launched in the Netherlands and resulted in the first clinical practice guideline, “Patient Documentation,” from the Royal Dutch Society for Physical Therapy (KNGF) (9). Since then, similar quality reporting programs have been implemented in the United States, Canada, Australia and Europe, and a number of books and articles have been published that address various aspects of the quality of care in general (3–6) and Dutch physiotherapy in particular (10–19). However, despite the increasing availability over the past decade of QIs designed to manage a variety of rheumatic and musculoskeletal diseases, the use of QIs in physiotherapy is still limited (20). Anno 2022, quality of physiotherapy still remains an important topic across various physiotherapy domains, including the domain Whiplash-Associated Disorders (WAD).

A particularly complex domain within physiotherapy is the quality of care in patients with WAD, a condition that is often presented to physiotherapists and remains difficult to manage. Whiplash injury is one of the most common traffic-related injuries (21) and is caused by acceleration-deceleration forces acting on the neck, head and torso (22, 23). The impact may result in lesions of cervical spine structures and effects on sensory, motor and mental functions, which in turn can lead to a variety of clinical manifestations, including neck pain, neck stiffness, headache, dizziness, tinnitus, paresthesia, loss of balance, loss of eye movement control, cognitive manifestations, and pain sensory disturbances indicative of sensitization of the peripheral and central nervous systems (24–30). These clinical manifestations are classified as WAD (31, 32). Worldwide, physiotherapy is one of the preferred treatment options for patients with WAD, especially when combined with other treatments such as medication (33). International data indicate that approximately 50% of people who sustain a whiplash injury will not recover and will continue to experience ongoing disability and pain 1 year after injury (34, 35). In addition to the poor prospects for recovery, poor treatment responses are another important issue (32, 36, 37). Today, many evidentiary gaps remain in terms of diagnostics, prognostics, and treatment, as well as concerning patient-related outcome measurements in patients with WAD (33, 38).

Clinical practice guidelines (CPGs) enable physiotherapists to assess (1) the extent to which physiotherapy management and assessment aligns with available research-based evidence, and (2) gaps in practice that need improvement. Routinely collected data (RCD) describing real practice populations, such as patients with WAD, can fill these evidentiary gaps and act an important driver of quality improvement and performance-based measurement (7, 39).

The present paper is an overview and synthesis of four recently published articles from our project group concerning the development and application of QIs in physiotherapy primary care. Using a routinely collected dataset, these papers explored quantitative measures of quality improvement in physiotherapy care in patients with WAD, based on the development and application of QIs embedded in the clinical reasoning process (40–43). Summarizing these papers, we introduce readers to the specific methodology of developing and applying QIs in patients with WAD in physiotherapy primary care. This approach can provide a framework and state of the art example for future QI research initiatives involving topics such as comparability of practitioners, inter-rater reliability, sensitivity to change and predictive validity.

## Clinical practice guidelines

Current national and international Clinical Practice Guidelines (CPGs) for patients with WAD are mainly based on systematic reviews and on primary studies of diagnostics, prognostics, and treatment outcomes (44–52). In general, comparable recommendations can be found across these guidelines, all of which are based on weak or moderate levels of evidence.

The Dutch CPG “Physiotherapy Management and WAD” was introduced in 2001 (44) and updated in 2002 (45) and 2016 (46). The content of Dutch CPGs is organized in accordance with a nine-step clinical reasoning process, in combination with the best available evidence and professional consensus. The clinical reasoning cycle is an internationally accepted concept to facilitate problem solving and decision making in the daily practice of physiotherapy. The transparency of this clinical reasoning process is considered a cornerstone of the quality of physiotherapy care (53).

Data are lacking on the complexity of the clinical reasoning process in patients with WAD (54). The lack of a detailed understanding of the clinical reasoning process related to various features of WAD may hamper the implementation of WAD-related CPGs in clinical practice and the delay improvement of physiotherapy quality in primary care.

## Defining quality indicators in healthcare using Donabedian's model

QIs have been defined as “measurable elements of practice performance for which there is evidence or consensus that they can be used to assess the quality of the care provided” (4, 5). They do not measure quality directly but are auxiliary variables that indirectly reflect the quality of care through ratios, thus one could also speak of quality-related indicators. Most initiatives to evaluate (improvement of) quality of care are consistent with Donabedian's model (55). Donabedian argued that the evaluation of context, process, outcome and structure indicators and their mutual relationships all provide a comparable picture of quality of care in different settings (55–57). Donabedian then postulated “relationships between the constructs of structure, process and outcome, based on the assumption that good structure should promote good processes and good processes should in turn promote good outcomes in a reciprocal pathway.”

“Structure” is defined as the professional and organizational resources associated with the provision of healthcare (e.g., availability of physiotherapy, equipment and staff training), “process” as the things done to and for the patient (e.g., practice referrals, clinical reasoning and decision), and “outcome” as the desired result of care provided by the health practitioner (e.g., a patient's functioning, and satisfaction with quality of care) (55–57). Context indicators were added to the postulated relationships and are indicators “that together constitute the complete context of an individual's life and living, and the background of an individual's health and health-related states in particular” (58).

## Development of quality indicators

The most commonly used method for the development of QIs is an iterated consensus rating procedure (5, 59, 60), such as the systematic RAND-modified Delphi method. By including independent expert comments and iterative feedback, this method results in a set of recommendations with good face validity and suitable for transcription into QIs (61).

The preferred method of QI development consists of five steps: (a) extraction of recommendations from CPGs, patient-related outcome measurements, and literature, particularly systematic reviews; (b) transformation of recommendations into QIs by phrasing them as the average degree (in %) to which patients were subjected to a methodically performed clinical reasoning process; (c) appraisal by an expert and user panel, including scoring of the set of QIs on a five-point Likert scale (1 = not at all to 5 = completely) based on acceptability, feasibility, clarity, and relevancy to the physiotherapy care process; (d) classification of process indicators into the nine steps of the clinical reasoning process; and (e) classification of outcome indicators in accordance with the International Classification of Functioning, Disability and Health (ICF) (58), e.g., body functions, activity and participation, as well as personal and environmental factors.

The methods used for indicator development in physiotherapy will be briefly explained by means of a recently published example concerning the quality of physiotherapy care in patients with WAD (40, 43). Two specialized physiotherapists independently extracted recommendations related to the nine steps of the physiotherapy clinical reasoning process, using sources including the Dutch CPG Physiotherapy Management and WAD (44, 45), the Quebec Task Force on WAD (31) and the updated Dutch CPG Neck Pain (including WAD) (46). Both physiotherapists were involved in the development of these CPGs. Following critical evaluation and checking for duplicates or overlap, 125 preselected items could be reduced to 96 and compared to current evidence (33, 38). Phrasing them as the average degree (in %) to which patients were subjected to a methodically performed clinical reasoning process, the 96 items were then transformed into a set of 28 QIs.

In the set of guideline-based QIs, quantified as percentages ranging from 0 to 100%, the number of times a QI was met was designated as numerator and the total number of patients was designated as denominator, thus *N* = 810 unless stated otherwise. We give some examples from the available WAD patient dataset in **Table 2**. For example, the numerator score for the number of patients subjected to previous medical imaging neck diagnostics (noted as yes) was 178/810 (QI = 21.9%); the extent to which physical examination objectives were formulated in agreement with patients' history taking (noted as yes) was 810/810 (QI = 100%), and the extent to which treatment goals were in agreement with the prognostic health profile and time phase since accident (noted as yes) was 529/810 (QI = 65.3%).

The level of research evidence for the formulated QIs, from levels I to IV, was determined based on a national consensus document (62), “with level I being the highest: level I = systematic review or >2 high-quality controlled trials or high-quality diagnostic studies or high-quality psychometric studies; level II = two high-quality controlled trials or high-quality diagnostic studies or high-quality psychometric studies; level III = high-quality non-controlled trials or low-quality diagnostic studies or low-quality psychometric studies; level IV = expert opinion and professional consensus or standard.” The level of evidence for most QIs was based on professional consensus (level IV) (40). Many reviews have called for further research to identify who does or does not respond to treatment. To date, clinical trials of WAD have not been able to identify factors associated with treatment response. Sterling et al. stated: “It would be fair to say that for musculoskeletal conditions, including neck pain and WAD, little progress has been made in this direction” (33).

## Set of quality indicators in steps of clinical reasoning in patients with WAD

Clinical reasoning has been defined “as a process in which the physiotherapist, interacting with the patient and significant others, structures meaning, goals and health management strategies based on scientific evidence, clinical data, client choices and professional judgment and knowledge” (53, 63, 64). The set of QIs are embedded in the nine steps of clinical reasoning, with the number of QIs assigned to each step indicated in parentheses: I: Patients' information (*n* = 2), II: History taking, (*n* = 7) III: Objectives of examination (*n* = 1), IV: Clinical examination (*n* = 5), V: Analysis and conclusion (*n* = 5), VI: Treatment plan (*n* = 3), VII: Treatment (*n* = 2), VIII: Evaluation (*n* = 5), and IX: Discharge (*n* = 2) (30–33).

Table 1 presents an overview of the complete set of QIs (*n* = 28) for the physiotherapy clinical reasoning process in patients with WAD (40–43) and also includes the items and the level of evidence per indicator.

**Table 1:** Quality indicators for physiotherapy care process in patients with Whiplash-Associated Disorders (WAD): steps of clinical reasoning, number of indicators, type of indicator, item measured, indicator, and level of evidence^*^ [adapted from Oostendorp et al. (40, 43)].

**Steps of clinical reasoning number of indicators**	**Type of indicator**	**Item**	**Indicator** **The average degree (in%) in which …**	Level of evidence^*^
**I. Patients information: 2 indicators (1–2)**				
	Process generic	Name, year of referral, referral, medical information	1. Patients information is shared.	IV
	Process specific	Information on referral lacking, period since accident, request for help	2. Patients request for help is noted.	IV
**II. History taking: 7 indicators (3–9)**				
IIa. Sociodemographic characteristics	Process generic	Age, gender, educational level, family status, employment status	3. Patients were subjected to a methodically performed history taking, and sociodemographic characteristics are noted.	IV
IIb. Accident-related information	Process specific	Location in vehicle, use of seatbelt, use of positioned headrest, anticipated collision, type of trauma, time of onset of whiplash-related complaints	4. Patients were subjected to a methodically performed history taking, and accident-related information is noted.	IV
IIc. Pre-existent functioning and health status	Process generic	Pre-existent activity limitations, participation problems, job-related problems	5. Patients were subjected to a methodically performed history taking, and pre-existent functioning is noted.	IV
	Process specific	Previous history of neck injury, pre-existent neck pain and/or stiffness, and/or irradiating arm pain, pre-existent pain else, comorbidity, relevant medication use	6. Patients were subjected to a methodically performed history taking, and pre-existent health status is noted.	IV
IId. Previous diagnostics and treatment	Process specific	Previous medical imaging neck diagnostics, cervical soft collar after trauma, pain medication, modalities of (manual) physiotherapy, recovery after previous treatment	7. Patients were subjected to a methodically performed history taking, and previous diagnostics and treatment are noted.	IV
IIe. Current health status and recovery rate since accident	Process generic	Impairments in musculoskeletal neck functions, activity limitations, participation problems, job-related problems	8. Patients were subjected to a methodically performed history taking, and current functioning are noted.	IV
	Process specific	Recovery rate since accident, type and number of complaints, type of signs and symptoms, inventory prognostic factors, pain medication, symptoms related to the presence of central sensitization	9. Patients were subjected to a methodically performed history taking, and recovery rate since accident, prognostic factors and the presence of central sensitization are asked and administrated.	IV
**III. Objectives of examination: 1 indicator (10)**				
IIIa. Objectives of musculoskeletal examination	Process specific	Examination objectives in agreement with patient's history taking and supplementary medical data, choice of clinical musculoskeletal, neurological and oto-neurological tests, and selection of psychological questionnaires	10. Examination objectives in agreement with patient's history are noted, and choice of clinical tests and psychological questionnaires is noted.	IV
IIIb. Objectives of neurological examination				
IIIc. Objectives of oto-neurological examination				
IIId. Objectives of psychological examination				
**IV. Clinical examination: 5 indicators (11–15)**				
IVa. Musculoskeletal examination	Process specific	Cervical testing (observation of posture, range of motion and palpation) in agreement with objectives of musculoskeletal examination	11. The results of clinical evaluation of cervical musculoskeletal functions testing are noted.	II–IV
IVb. Neurological examination	Process specific	Testing of sensory functions and pain, muscles functions, reflexes and coordination, and testing of cranial nerve functions (partly incorporated in oto-neurological examination, particularly trigeminal nerve) in agreement with objectives of neurological examination	12. The results of clinical evaluation of neurological functions are noted.	IV
IVc. Oto-neurological examination	Process specific	Standing and gait testing, dizziness test, positional testing, eyes movement test in agreement with objectives of oto-neurological examination	13. The results of clinical evaluation of equilibrium and dizziness/vertigo are noted.	IV
IVd. Psychological examination	Process specific	Observation of pain behavior, and psychological questionnaires (Fear- Avoidance Beliefs Questionnaire—FABQ—and Pain Coping Inventory -PCI)	14. The results of examination of psychological functions and tests are noted.	II–IV
	Process specific	Presence of central sensitization	15. Presence of central sensitization is noted.	IV
**V. Analysis and conclusion of diagnostic process: 1 indicator (16)**				
	Process specific	Classification Whiplash-Associated Disorders, time phase since accident, recovery in time since accident, determination of health profile A/B/C, prognostic factors, use of questionnaires, referral to GP in case if insufficient or no results expected, indication physiotherapy	16. Individual health profile addressed to the whiplash injury since accident, an indication of treatment prognosis, and an indication for physiotherapy have been established and are noted.	II–IV
**VI. Treatment plan: 3 indicators (17–19)**				
Via. Profile-based treatment goals	Process specific	Main treatment goals in different time phases since accident and in agreement with individual health profile and patient	17. Treatment goals are methodically determined and noted in agreement with individual prognostic health profile, time phase since accident, and with patient.	IV
VIb. Duration of treatment period and number of sessions	Process specific	Prognostic duration of treatment period and prognostic number of treatment sessions	18. Prognostic treatment period and number of treatment sessions are noted.	IV
Vic. Pretreatment measurements	Process specific	Pre-treatment measures pain (VAS) and functioning (NDI)	19. Pre-treatment scores VAS and NDI are measured and noted.	I
**VII. Treatment: 2 indicators (20–21)**				
VIIa. Best evidence treatment options in agreement with treatment goals	Process specific	Physiotherapy modalities with best available evidence in different time phases since accident in agreement with patient profile and treatment goals	20. Physiotherapy modalities in agreement with treatment goals in time phases since accident and health profile, and with best available evidence are applied and noted.	II
VIIb. Side effects	Process generic	Check for side effects	21. Treatment effects and side effects are noted in patient's record.	IV
**VIII. Evaluation: 5 indicators (22–26)**				
VIIIa. Evaluation during treatment	Process specific	Perceived result per treatment goal, regular and systematic evaluation and, if necessary, adjustment	22. A methodically performed evaluation of treatment goals and	IV
		of treatment goals and treatment modalities, contact physician if insufficient treatment result	treatment modalities are noted.	
VIIIb. Final evaluation	Outcome Generic	Final subjective and objective evaluation of treatment goals, post-treatment measures (pain (VAS) and functioning (NDI), global perceived	23. Reached treatment goals and returned to work are subjectively evaluated and noted.	IV
		effect (GPE), return to work, duration of treatment period and number of treatment sessions at the end of total treatment	24. Post-treatment scores [pain (VAS) and functioning (NDI)] are measured and noted.	I
			25. Global perceived effect is measured and noted.	II
			26. Duration of treatment period and number of treatment sessions are noted.	IV
**IX. Discharge: 2 indicators (27–28)**				
	Process generic	Reason for discharge, written report to physician in copy to patient	27. A final report is written and noted.	IV
	Process specific	If necessary, arrangement of aftercare	28. Aftercare is arranged	IV

* Levels of evidence: I, systematic review or >2 high-quality controlled trials or high-quality diagnostic studies or high-quality psychometric studies; II, two high quality-controlled trials or high-quality diagnostic studies or high-quality psychometric studies; III, high quality non-controlled trials or low-quality diagnostic studies or low-quality psychometric studies; IV, experts' opinion and professional consensus or standard.

## Routinely collected dataset of patients with WAD

The first WAD pen and paper patient record was introduced in 1996 in two primary care physiotherapy practices based on the first CPG Patient Documentation (9). The Medical Ethics Committee of Radboud University Medical Center Nijmegen, the Netherlands, waived in writing the requirement for ethical approval as the dataset involved routinely collected data that represented no extra burden for participating patients (www.ru.nl/rdm/collecting-data/informed-consent-ethics-committees).

The participating physiotherapists received updates in accordance with adjustments to the content of the most recent CPG and the adapted patient record files, explained in 3 h meetings in 2001, 2002, 2009, and 2016, respectively. They also received instructions on how to score items for each step of the clinical reasoning process. All patient records were archived and relevant characteristics of the dataset are presented below and in Table 2, ordered according to the diagnostic, therapeutic and evaluative steps of clinical reasoning.

**Table 2:** Item scores per indicator of diagnostic clinical reasoning process in patients with Whiplash-Associated Disorders (WAD) [adapted from Oostendorp et al. (40)] N = 810; n (%) unless otherwise stated.

**Steps of diagnostic clinical reasoning**	**Total**
**process**	**N = 810**
	**n (%)**
**I. Patient's information**	
**Indicator 1—Patient's information**	
Year of referral	810 (100.0)
**Referral**	
General physician	549 (67.8)
Medical specialist	164 (20.2)
Self-referral	97 (12.0)
**Indicator 2—Request for care**	
Information on referral lacking	148 (18.3)
**Time phase since accident**	
Phase 1 (<7 days)	19 (2.3)
Phase 2 (1–3 weeks)	140 (17.3)
Phase 3 (4–6 weeks)	192 (23.7)
Phase 4 (7–12 week)	183 (22.6)
Phase 5 (3–6 months)	155 (19.1)
Phase 6 (>6 months)	121 (14.9)
**Request for care**	
Reducing pain	759 (93.7)
+ Explaining consequences of whiplash	12 (1.5)
+ Improving functions	38 (4.7)
+ Increasing activities and participation	1 (0.1)
**II. History taking**	
**Indicator 3—Sociodemographic characteristics**	
Age (year) (mean; sd)	43.0 (12.6)
Gender (female)	586 (72.3)
Educational level^*^(low)	450 (55.6)
Employment status (employed)	510 (62.0)
**Indicator 4—Accident characteristics**	
Direction of impact (back)	512 (63.2)
Anticipated collision (no)	583 (72.0)
**Type of trauma**	
Neck trauma without head trauma	572 (70.6)
Neck trauma with head trauma	198 (24.4)
Other trauma	40 (4.9)
Unknown	–
**Time of onset whiplash-related complaints**	
Immediately	145 (17.9)
≤2 days	556 (68.9)
3–7 days	109 (13.5)
>1 week	–
**Indicator 5—Preexistent functioning**	
**Functioning problems**	
Activity limitation (yes)	125 (15.4)
Participation problems (yes)	109 (13.5)
Job-related problems (yes)	93 (11.5)
**Indicator 6—Preexistent health status**	
Relevant medication use (yes)	107 (13.2)
Previous history of neck injury (yes)	81 (10.0)
Previous neck pain and stiffness (yes)	144 (17.8)
Pain else (yes)	150 (15.8)
**Indicator 7—Previous diagnostics and treatment**	
Medical imaging neck diagnostics (yes)	178 (22.0)
Cervical soft collar (yes)	514 (63.4)
Weeks (mean; sd)	3.9 (2.0)
Pain medication (yes)	369 (45.6)
(Manual) physiotherapy (yes)	332 (40.0)
**Recovery after previous treatment**	
Fully recovered	**–**
Partially recovered	43 (5.3)
Stabilization	263 (32.5)
Deterioration	314 (38.8)
Inestimable	190 (23.5)
**Indicator 8—Current health status**	
**Functioning problems**	
Impairments in musculoskeletal neck functions (yes)	810 (100.0)
Activity limitation (yes)	688 (84.9)
Participation problems (yes)	712 (87.9)
Job-related problems (yes)	312 (38.5)
Pain medication (yes)	242 (29.9)
**Type and number of complaints**	
≤3: neck pain, stiffness, decreased ROM^*^	6 (0.7)
4–6: + dizziness, headache and tinnitus	374 (46.2)
7–9: + cognitive impairments	424 (52.3)
>9: + rest	6 (0.7)
**Indicator 9—Prognostic factors and recovery rate**	
**Inventory prognostic factors (modified Waddell's sign; n = 575) ^#^**	
≤3	45 (7.8)
>3	530 (92.2)
**Use of coping**	
Active	329 (40.7)
Passive	443 (57.7)
Inestimable	38 (3.7)
**Fear of avoidance**	
Yes	467 (57.7)
No	146 (18.2)
Inestimable	197 (24.3)
**Presence of signs of central sensitization (n = 149)**	
Yes	66 (44.3)
No	7 (4.7)
Inestimable	76 (51.0)
**Recovery rate since accident**	
Normal	–
Delayed	441 (54.4)
Inestimable	369 (45.6)
**III. Objectives of examination**	
**Indicator 10—Examination objectives in agreement with history—choice**	
**of tests**	
Objectives of musculoskeletal examination (yes)	810 (100.0)
Objectives of neurological examination (yes)	136 (16.8)
Objectives of oto-neurological examination (n = 621) (yes)	376 (60.5)
Objectives of psychological examination (n = 621) (yes)	577 (92.9)
**IV. Clinical examination**	
**Indicator 11—Results of musculoskeletal tests**	
**Musculoskeletal examination**	
Observation of posture (yes)	810 (100.0)
Active examination of neck function (yes)	810 (100.0)
Passive examination of neck function (yes)	810 (100.0)
Palpation of tender points (yes)	810 (100.0)
**Indicator 12—Results of neurological tests**	
**Neurological examination**	
Sensory testing	136 (16.8)
Motor testing	130 (16.0)
Reflex testing	130 (16.0)
Coordination testing	91 (11.2)
**Indicator 13—Results of oto-neurological tests**	
**Oto-neurological examination (n = 621)**	
Standing tests	346 (55.7)
Walking tests	366 (58.9)
Dizziness tests	376 (60.5)
Nystagmus tests	376 (60.5)
Dix-Hallpike test	21 (3.4)
**Indicator 14—Results of psychological tests**	
**Psychological examination**	
Observation of pain behavior and fear avoidance (n = 621)	577 (92.9)
Use of coping questionnaire (n = 523)^##^	495 (94.6)
Use of fear avoidance questionnaire (n = 523)^##^	495 (94.6)
**Indicator 15—Presence of central sensitization**	
Presence of signs of central sensitization (n = 149) (yes)	47 (41.5)
**V. Analysis and conclusion of diagnostic process**	
**Indicator 16—WAD classification—indication—prognosis**	
**Classification WAD *^###^***	
WAD 0	–
WAD 1	123 (15.2)
WAD 2	555 (68.5)
WAD 3	132 (16.3)
WAD 4	–
**Time phase since accident**	
>7 days	19 (2.3)
1–3 weeks	140 (17.3)
4–6 weeks	192 (23.7)
7–12 weeks	183 (22.6)
3–6 months	155 (19.1)
>6 months	121 (14.9)
**Recovery rate since accident**	
Normal	–
Delayed	441 (54.4)
Inestimable	369 (45.6)
**Determination of health profile *^####^***	
Profile A	–
Profile B	369 (45.6)
Profile C	441 (54.4)
**Prognostic factors related to recovery**	
Observation pain behavior (n = 621) (yes)	577 (92.9)
Modified Waddell's sign (n = 575) (>3)^#^	530 (92.2)
Use of passive coping (yes)	443 (54.7)
Fear avoidance (yes)	467 (57.7)
Presence of signs of central sensitization (n = 149) (yes)	47 (41.5)
Consultation referring physician about indication	232 (28.6)
**Indication physiotherapy**	
Yes	632 (78.0)
No	–
Doubtful	178 (22.0)

* Educational level: low, advanced, high. ^#^Modified Waddell's signs: tenderness, stimulation, cervical Range of Motion (ROM), regional disturbance and overreaction.

## Psychological questionnaires: Fear Avoidance Beliefs Questionnaire (FABQ) and Pain Coping Inventory (PCI).

### Classification WAD: Whiplash-Associated Disorders: WAD 0: no neck symptoms, no physical sign(s); WAD 1: neck pain, stiffness or tenderness only, no physical sign(s); WAD 2: neck symptoms and musculoskeletal sign(s); WAD 3: neck symptoms and neurological sign(s); WAD 4: neck symptoms and fracture or dislocation.

#### Health Profile: Profile A: normal recovery, low intensity of pain, decreasing pain, increasing activities; Profile B: inestimable recovery, middle intensity of pain, persistent pain, persistent activity limitations; Profile C: delayed recovery, high intensity of pain, increasing pain, decreasing activities.

Sociodemographic characteristics of the total group (*N* = 810) are presented in Table 2. Patient's mean age was 43.0 years (SD 12.6) and 586 (72.3%) were female. The most frequent accident-related characteristics were direction of impact (back *n* = 512; 63.2%), neck trauma without head trauma (*n* = 572; 70.6%), and onset of whiplash-related complaints within 3 days (*n* = 556; 68.9%).

### Diagnostic steps of the clinical reasoning process

An overview of the item scores per step of the diagnostic part of the clinical reasoning process is presented in Table 2, adapted from Oostendorp et al. (40).

Most patients (*n* = 555; 68.5%) were classified as WAD-2, with a delayed recovery (*n* = 441; 54.4%), and were referred 7 weeks to >6 months after the accident (*n* = 459; 56.7%). Eighty-one patients (10.0%) reported a previous history of neck injury, and 144 patients (17.8%) a history of neck pain and stiffness. Half of the patients had been previously treated with several interventions, such as pain medication (*n* = 369; 45.6%), cervical soft collar (*n* = 514; 63.4%) or (manual) physiotherapy (*n* = 332; 40.0%). No patients were fully recovered and 43 (5.3%) were partially recovered. The results of earlier treatment were inestimable in 190 patients (23.5%), while in 263 patients (32.5%) a stabilization in functioning was estimated, and 314 patients (38.8%) showed a deterioration in functioning.

A number of potentially negative prognostic factors for recovery were found, including pain intensity (high level of pain intensity in the acute phase), low level of functioning, recovery rate since accident (inestimable [*n* = 369; 45.6%] and delayed recovery [*n* = 441; 54.4%]), modified Waddell's non-organic physical signs (>3: *n* = 530; 92.2%), risk for passive coping (*n* = 443; 54.7%) and risk for fear avoidance (*n* = 467; 57.7%). The prognostic factors were summarized in three prognostic recovery profiles, ranging from a positive profile (profile A) to a negative profile (profile C). Profile B is characterized by both positive and negative factors, making it difficult to estimate (inestimable) the chance of recovery (Profile B). No patients were classified in prognostic health profile A, 369 (45.6%) in profile B and 441 patients (54.4%) in profile C.

In conclusion, all patients developed persistent symptoms ranging from mild to severe pain and disability following their accident. They were referred more than 3 months after their accident (most recovery occurs within the first 3 months after which time the condition tends to plateau), and a majority of patients used a cervical collar (brace) during 4 weeks on average. Around half of the patients showed a delayed recovery rate following their accident, and the remaining group had an inestimable recovery time.

Based on clinical analysis and consequent conclusions, and following consultation with the patient and the patient's referring physician concerning an indication for physiotherapy, physiotherapy was possibly indicated in 178 patients (22.0%) and definitely indicated in 632 patients (78.0%), classified in phases from 1 to 6.

### Therapeutic and evaluative steps of the clinical reasoning process

An overview of the item scores per step of the therapeutic and evaluative part of the clinical reasoning process for the total group (*N* = 810) is presented in Table 3, adapted from Oostendorp et al. (40, 41).

**Table 3:** Item scores of therapeutic and evaluative process of clinical reasoning process in patients with Whiplash-Associated Disorders (WAD) [adapted from Oostendorp et al. (40)] N = 810; n (%) unless otherwise stated.

**Steps of therapeutic and evaluative clinical reasoning (steps VI–IX)**	**Total**
	**N = 810**
	**n (%)**
**VI. Treatment plan**	
**Indicator 17—treatment goals**	
Phase 1: <7 days: reducing pain; providing information and explaining the functioning consequences and underlying pain mechanisms (n = 19) (yes/no)	11 (57.9)/8 (42.1)
Phase2: 1–3 weeks: see Phase 1 + improving functions (n = 140) (yes/no)	82 (58.6)/58 (41.4)
Phase 3a (inestimable recovery): 4–6 weeks: see Phase 2 + increasing activities and participation (n = 17) (yes/no)	12 (70.6)/5 (29.4)
Phase 3b (delayed recovery): 4–6 weeks: explaining underlying pain mechanisms, improving active coping, decreasing fear avoidance, increasing physical loadability, increasing activities and participation (n = 175) (yes/no)	96 (54.9)/79 (45.1)
Phase 4a (inestimable recovery): 7–12 weeks: see Phase 3a + minimizing delay in work participation (n = 8) (yes/no)	5 (62.5)/3 (37.5)
Phase 4b (delayed recovery): 7–12 weeks: see Phase 3b (n = 175) (yes = y; no)	124 (70.9)/51 (29.1)
Phase 5 (chronic): 3–6 months: see Phase 3b (n = 155) (yes/no)	128 (82.6)/27 (17.4)
Phase 6 (chronic): > 6 months: see Phase 3b (n = 121) (yes/no)	71 (58.7)/50 (41.3)
**Indicator 18–Pre-estimated treatment period and number of sessions**	
**Prognostic duration of treatment period**	
<3 months	64 (7.9)
4–6 months	230 (28.4)
>6 months	516 (63.7)
**Prognostic number of treatment sessions**	
1–10 sessions	78 (.9.6)
11–15 sessions	253 (31.2)
16–20 sessions	313 (38.6)
>20 sessions	166 (20.5)
**Indicator 19—Pre-treatment scores pain and functioning**	
Pre-treatment measures pain [Visual Analogue Scale (VAS): 0–100] and functioning [Neck Disability Index (NDI): 0–50] (n = 523) (yes/no)	495 (94.6)/28 (5.4)
Treatment plan in agreement with patient (yes)	810 (100.0)
**VII. Treatment**	
**Indicator 20—Treatment modalities**	
Phase 1: Education, coaching, active exercise therapy (n = 11) (yes/no)	9 (81.8)/ 2 (18.2
Phase 2: See Phase 1 + cervical soft collar (<1 week), massage therapy (<2 weeks) (n = 82) (yes/no)	67 (81.7)/ 15 (18.3)
Phase 3a: See Phase 1 + physical loading exercise therapy (n = 12) (yes/no)	10 (83.3)/ 2 (16.7)
Phase 3b: Pain education, exercise therapy based on cognitive and physical principles (n = 96) (yes/no)	80 (83.3)/ 16 (16.7)
Phase 4a: See Phase 3a + graded activity (n = 5) (yes/no)	3 (60.0)/ 2 (40.0)
Phase 4b: See Phase 3b + graded exposure (n = 124) (yes/no)	107 (86.3)/ 16 (13.7)
Phase 5: See Phase 4b (n = 128) (yes/no)	110 (85.9)/ 18 (14.1)
Phase 6: See Phase 5 (n = 71) (yes/no)	56 (78.9)/ 15 (21.1)
**Indicator 21—Side effects**	
Check for treatment side or adverse effects (yes)	810 (100.0)
**VIII. Evaluation**	
**Indicator 22—Evaluation during treatment**	
Evaluation during treatment process (yes)	790 (97.5)
If necessary, adjustment of treatment goals and modalities (yes)	185 (22.8)
Contact physician if insufficient treatment result (yes)	247 (30.5)
**Indicator 23—Final evaluation**	
Treatment goals (yes)	810 (100.0)
**Indicator 24—Post-treatment scores pain and functioning**	
Post-treatment measures pain [Visual Analogue Scale (VAS): 0–100] and functioning [Neck Disability Index (NDI): 0–50] (n = 523) (yes/no)	495 (94.6)/ 28 (5.4)
**Indicator 25—Global perceived effect**	
Evaluation by Global Perceived Effect (GPE 0–7) (n = 523) (yes/no)	495 (94.6)/ 28 (5.4)
**Indicator 26—Final evaluation treatment period and number of sessions**	
**Duration of treatment period**	
2–3 months	280 (34.6)
4–6 months	501 (61.9)
>6 months	29 (3.6)
**Number of treatment sessions**	
<5	2 (0.2)
5–10	10 (1.2)
11–15	329 (40.6)
16–20	405 (50.0)
>20	64 (7.9)
**IX. Discharge**	
**Indicator 27—Reason for discharge and report**	
Reason for discharge (yes)	810 (100.0)
Written report (yes)	810 (100.0)
**Indicator 28—Aftercare**	
Arrangement of aftercare (since 2003; n = 457) (yes)	151 (33.0)

The settings of treatment goals were in agreement with the prognostic health profiles and the time phases 1–6 since the accident in 529 (65.3%) of 810 patients but in disagreement in 281 patients (34.7%). Physiotherapy modalities were in agreement with treatment goals and best available evidence in 366 (69.2%) of 529 patients but in disagreement in 163 patients (30.8%). The pre-estimated treatment duration was >6 months in 516 patients (63.7%) and the pre-estimated number of treatment sessions was ≥16 in 479 patients (59.1%). Patient-related outcome measurements were available in 523 patients. Intensity of pain was reduced to ≤30 (Visual Analogue Scale [VAS] 0–100) in 301 patients (59.3%) and functioning was improved to ≤14 (Neck Disability Index [NDI] 0–50) in 191 patients (36.5%). Approximately half of the patients (*n* = 241; 46.1%) were improved based on the global perceived improvement scale (GPE from “improved” [very good, good and fairly improved], to “no change” [same as before] and “worse” [worse and much worse]).

The treatment plan for about two thirds of patients was in line with the time phase after accident and the prognostic profile. However, this was not the case in around one third of patients. Therefore, if patients were assigned the correct time phases and prognostic profiles, the composition of treatment modalities suits the treatment plan in more than two thirds of cases, but is discordant with the treatment plan in around one third of patients. In conclusion, we can therefore safely conclude that there is abundant room for data-driven quality improvement of physiotherapy management in patients with WAD.

Despite the poor prospects for functional recovery at initial contact with the physiotherapist, about half of the patients rated the perceived treatment effect as “improved,” ranging from very improved to fairly improved, and more than half of all patients rated a reduction in the intensity of pain (to minimal pain), while in around one third of patients functioning was improved to “optimal functioning.” These patient-related outcomes underline the fact that around 50% of patients were not recovered at 1 year and experience ongoing disability and pain after a whiplash-related injury.

In contrast to longitudinal studies (34, 65–70), the data presented here only include data gathered during the treatment episode, without additional follow-up. Within these limitations, about half of the patients improved while the other half were categorized as “no change” or “worse,” without meaningful differences related to the year of treatment or the phase after whiplash-related injury. Based on the results of longitudinal studies of functional recovery after whiplash-related injury, it seems unlikely that recovery rates of the described patients will improve substantially in the future. International data also indicate that ~50% of people involved in a whiplash-causing accident will not recover and will continue to experience ongoing activity limitations, participation problems, and long-term neck pain (34, 35).

## From dataset to quality indicator percentages

The formula for percentages of each QI is used as a sum score of the percentages of the dichotomized items, divided by the number of items per indicator. The overall QI scores are used as unweighted sum scores of the percentages per year as numerator and the number of years (*n* = 16) as denominator. The QIs per step of the clinical reasoning process are expressed as mean percentages (including standard deviation, minimum and maximum, and median).

To facilitate interpretation of performance targets, QI percentage scores were classified from “negligible” (0%) to “excellent” (100%) or from “excellent (0%) to “negligible” (100%), depending on the direction of the indicator (0–20% “negligible” or “excellent”; 21–40% “weak” or “good”; 41–60% “sufficient”; 61–80% “good” or “weak”; 81–100% “excellent or ‘negligible'). For instance, the direction of inventory of prognostics factors” (Indicator 9) was from 0% (negligible) to 100% (excellent) and the direction of previous diagnostics (Indicator 7) from 0% (excellent) to 100% (negligible). Most indicators were categorized from 100 to 0% (from excellent to negligible), and three indicators went from 100 to 0% (from negligible to excellent) (40).

A desired performance target regarding quality of physiotherapy care can be determined in consultation with different stakeholders. In consultation with physiotherapists working in primary care. the Dutch Royal Association for Physical Therapy (KNGF) has established target standards for QIs related to steps of the clinical reasoning process (≥70% and ≤30% depending on the desired direction of the indicator. In the current context, the performance target was set to ≥70% for 23 indicators (QIs 1–4, 8–15, 17–27) and to ≤30% for 3 indicators (QIs 5–7), while two indicators (QIs 16 and 28) remained non-defined due to their innovative character. See Table 4. The indicator “the number of patients in whom symptoms related to central sensitization are present” needs further elaboration. There is growing evidence to suggest that chronic WAD is associated with impairments in generalized sensory hypersensitivity as a result of sensitized pathways within the central nervous system (24, 25).

**Table 4:** Long term evaluation of quality of clinical reasoning process of physiotherapy in patients with Whiplash-Associated Disorders (*n* = 810 unless otherwise stated).

**Clinical reasoning process (Number of indicators)**	**Mean**	**SD**	**Minimum**	**Median**	**Maximum**	**Performance target** **+ (≥70% or ≤30%)** – **(<70% or >30%)**
I. Patient's information (n = 2) ^*^/^**^						
Indicator 1: patient's information	84.2	4.8	72.7	84.7	92.5	+ (≥70%)
Indicator 2: patient's request for help	85.5	4.0	79.8	84.4	95.8	+ (≥70%)
II. History taking (n = 7) ^*^/^**^						
Indicator 3: sociodemographic characteristics	92.7	4.2	84.2	92.7	100.0	+ (≥70%)
Indicator 4: accident-related information	80.2	5.1	71.4	79.4	90.8	+ (≥70%)
Indicator 5: pre-existent functioning	15.4	4.9	7.8	15.4	23.0	+ (≥70%)
Indicator 6: pre-existent health status before injury	14.5	6.1	3.9	15.2	24.4	+ (≥70%)
Indicator 7: previous diagnostics and treatment	46.7	13.4	33.0	42.1	78.6	– (>30%)
Indicator 8: current health status/functioning in ICF terms	100.0	0.0	100.0	100.0	100.0	+ (≥70%)
Indicator 9: recovery since accident and prognostic factors	70.7	6.8	50.8	72.2	79.4	+ (≥70%)
III. Objectives of examination (n = 1) ^*^/^**^						
Indicator 10: objectives of examination	65.5	7.9	50.5	64.4	76.7	– (<70%)
IV. Clinical examination (n = 5) ^*^/^**^						
Indicator 11: musculoskeletal examination	100.0	0.0	100.0	100.0	100.0	+ (≥70%)
Indicator 12: neurological examination	81.4	16.3	51.4	82.7	100	+ (≥70%)
Indicator 13: oto-neurological examination (n = 621)	67.0	20.5	26.6	75.9	88.0	– (<70%)
Indicator 14: psychological examination: observation, psychological questionnaires (n = 621)	86.2	15.1	32.6	100	100	+ (≥70%)
Indicator 15: presence of central sensitization (n = 149)	46.5	7.5	37.9	49.8	51.8	Non-defined
V. Analysis and conclusion diagnostic process (n = 1) ^*^/^**^						
Indicator 16: analysis and conclusion of diagnostic process	71.2	14.0	48.8	80.7	88.2	+ (≥70%)
VI. Treatment plan (n = 3) ^*^/^**^						
Indicator 17: treatment goals	89.0	4.3	78.7	90.2	94.0	+ (≥70%)
Indicator 18; prognostics of treatment period and sessions	76.0	12.2	38.5	79.4	91.8	+ (≥70%)
Indicator 19: Pre-treatment scores Pain (VAS) and functioning (NDI) (n = 523)	100.0	0.0	100.0	100.0	100.0	+ (≥70%)
VII. Treatment (n = 2) ^*^/^**^						
Indicator 20: physiotherapy modalities	69.2	10.2	39.9	70.0	83.0	– (<70%)
Indicator 21: side effects	100.0	0.0	100.0	100.0	100.0	+ (≥70%)
VIII. Evaluation (n = 5) ^*^/^**^						
Indicator 22: evaluation during treatment	76.9	6.0	64.8	77.1	84.1	+ (≥70%)
Indicator 23: subjective end evaluation treatment goals	91.2	4.7	83.9	91.5	100.0	+ (≥70%)
Indicator 24: objective end evaluation post-treatment pain (VAS) and functioning (NDI) (n = 523)	94.6	13.5	57.6	100.0	100.0	+ (≥70%)
Indicator 25: global perceived effect (n = 523)	95.7	13.4	57.6	100.0	100.0	+ (≥70%)
Indicator 26: duration treatment period and number treatment sessions	100.0	0.0	100.0	100.0	100.0	+ (≥70%)
IX. Discharge (n = 2) ^*^/^**^						
Indicator 27: final report of discharge	100.0	0.0	100.0	100.0	100.0	+ (≥70%)
Indicator 28: after care (n = 151)	32.4	11.0	12.0	34.9	44.8	Non-defined

* Full description of quality indicators: see Table 1 [adapted from Oostendorp et al. (40, 43)].

** Full description of type and scores of variables per indicator: see Table 2 [adapted from Oostendorp et al. (40)] and Table 3 [adapted from Oostendorp et al. (40)].

## Application of WAD-related quality indicators in the clinical reasoning process

Using the set of QIs together with our routinely collected dataset, the quality of physiotherapy care in patients with WAD was evaluated. To translate data into QI scores expressed as frequencies, we formulated algorithms that followed the process of clinical reasoning in patients with WAD, thus allowing target performance to be defined.

The percentages of QIs per step of the clinical reasoning process are presented in Table 4.

Four QIs (indicators 7, 10, 13, 20) did not meet the performance target, the target performance of two QIs (indicators 15 and 28) was non-defined as discussed above, while 22 QIs met the performance targets of ≥70% or ≤30% over a period of 16 years.

The number of positively-assessed QIs for performance targets continued to improve over a period of 16 years in which the data were collected. The most striking quality improvements were seen in the clinical examination (based on the objectives of examination), the analysis and conclusion of the diagnostic process in the transition to treatment plan and treatment, and in the frequency of use of patient-related outcome measurements such as pain intensity, functioning and global perceived effect (GPE). However, there is still room for improvement in clinical practice.

## Suitability of routinely collected data for quality evaluation of physiotherapy care

RCD offer several advantages. Data collection under real-life practice conditions maximizes representativeness and generalizability, minimizes costs and effort, and allows the capture of information from large populations and many clinical practices over long periods (7, 71). However, these advantages should be viewed with caution, as errors and biases due to incomplete registration can interfere with results (7, 71, 72). Registry data are generally only visible within the local practice and are not routinely used to improve quality of physiotherapy care on a national level. To use RCD properly, certain challenges and barriers must be overcome. Reluctance of healthcare providers to supply data, poor integration in daily practice, limited availability of skills and lack of funding have been identified as the most frequent barriers to use (7).

As many evidentiary gaps persist concerning the prognostics, diagnostics and treatment of patients with WAD, the use of centralized, on-going RCD generally represents a useful alternative approach to understanding the quality of physiotherapy care. RCD on physiotherapy management in patients at different phases of WAD may provide a more complete view of the clinical reasoning process and a more comprehensive and realistic view of routine practice compared to data gathered during an RCT. In the majority of RCTs (*n* = 122) involving patients with non-specific neck pain the reporting of the clinical reasoning process was incomplete, specifically in the diagnostic aspect of the process, with only 6% of the RCTs including a complete diagnostic process (73).

As a counterbalance to the overreliance on RCTs as the highest level of evidence establishing treatment effectiveness, there is increasing interest in clinical research that includes a broad selection of patients, has less strict inclusion and exclusion criteria and uses patient-reported outcomes (7, 71). The number of real-life studies has been rapidly growing in different areas of medicine like respiratory medicine (74). Nevertheless, few physiotherapy studies utilizing RCD have been published to date (71). In an effort to improve assessment of the quality of real-world studies, the RECORD statement (Reporting of studies Conducted using Observational Routinely-collected health Data) was recently formulated (75). The RECORD statement is a checklist of items, including codes to identify participants and to classify patient characteristics, exposures, confounders and outcomes. Most items covered by the RECORD statement were included in our observational studies using RCD in patients with WAD (40, 42). We anticipate that the RCD underlying our observational study could plausibly act as preliminary real-world evidence concerning (manual) physiotherapy management and WAD, and could be used to improve the design of future data-driven clinical improvement studies' (76, 77).

The data on WAD patients described here were routinely collected over a period of 16 years in a large population of patients with WAD, using broad inclusion and limited exclusion criteria. These data therefore reflect the heterogeneity of real practice populations under routine care conditions, conditions that differ from the artificial situation of an RCT. To the best of our knowledge, this is the only example of the use of RCD in the (manual) physiotherapy management of WAD patients.

## Concluding remarks

To our knowledge, the set of QIs discussed here is the first set to be developed specifically for measurement of the quality of physiotherapy care in patients with WAD. The good face and content validity of this set indicates suitability for application in primary care physiotherapy practice. Further research (5, 6) will be needed to provide evidence of acceptability, reliability, sensitivity to change, and predictive validity of this set of QIs of physiotherapy care in patients with WAD.

The set of QIs described here, embedded in a clinical reasoning process for patients with WAD, can be used as a starting point for research on the clinimetric properties that measure the sensitivity to change in quality of primary care physiotherapy in patients with WAD.

The combination of a variety of evidence regarding primary care physiotherapy management of patients with WAD and neck pain will provide a broader view of the clinical reasoning process, and hopefully promote a more comprehensive and realistic view of the (improvement of) quality of routine practice when compared to data gathered exclusively during an RCT (or even pragmatic clinical trials).

We would argue that routinely collected data can aid improvement of the quality of (manual) physiotherapy through benchmarking, personalization, and continued education, not only in patients with WAD, but also in other musculoskeletal (pain) conditions. Furthermore, international consensus on a set of QIs embedded in the physiotherapy clinical reasoning process, as well as on performance targets and scoring procedures, would help considerably in improving comparisons between studies of physiotherapy care quality in patients with WAD. We urge policy makers, professional Associations, Clinicians and Researchers Across the Globe to Consider Investing Resources in the development and application of QIs for monitoring and improving (physiotherapy) care for patients with WAD.

## Data availability statement

The original contributions presented in the study are included in the article/supplementary material, further inquiries can be directed to the corresponding author/s.

## Author contributions

All authors contributed to drafting and revising the article, gave final approval of the version to be published, and agree to be accountable for all aspects of the work.

## Conflict of interest

The authors declare that the research was conducted in the absence of any commercial or financial relationships that could be construed as a potential conflict of interest.

## Publisher's note

All claims expressed in this article are solely those of the authors and do not necessarily represent those of their affiliated organizations, or those of the publisher, the editors and the reviewers. Any product that may be evaluated in this article, or claim that may be made by its manufacturer, is not guaranteed or endorsed by the publisher.
